# Cognitive simulation for the procedural skills learning of medical students: A systematic review.

**DOI:** 10.12688/mep.21205.2

**Published:** 2025-11-16

**Authors:** Khang Duy Ricky Le, Sarah Munday, Asha Taurins, Kellie Britt, Cameron Shaw

**Affiliations:** 1 School of Medicine, Deakin University Faculty of Health, Geelong, Victoria, 3220, Australia; 2 Department of General Surgical Specialities, The Royal Melbourne Hospital, Parkville, Victoria, 3052, Australia; 3 Department of Paediatrics, Monash Health, Clayton, Victoria, 3168, Australia; 4 Monash Bioethics Centre, Monash University Faculty of Arts, Clayton, Victoria, 3168, Australia; 5 Hobart Clinical School, University of Tasmania School of Medicine, Hobart, Tasmania, 7001, Australia

**Keywords:** Mental rehearsal, mental imagery, cognitive simulation, simulation-based education, simulation-based teaching, medical student, medical education, procedural skills, clinical skills.

## Abstract

**Background:**

Cognitive simulation, where individuals deliberately rehearse procedural tasks using the mind without physical action, has been demonstrated to improve technical skills across various disciplines such as aviation and elite sport. These practices however are used variably in medical education. Therefore, the efficacy and value of cognitive simulation in improving procedural skills of medical students remains unknown.

**Methods:**

Medline, Embase, CINAHL, Emcare and the World Health Organisation (WHO) International Clinical Trials Registry Platform databases were searched for articles that explored cognitive simulation approaches for the procedural skills development of medical students. Outcomes of interest included improvements in technical skill and proficiency, non-technical skills such as confidence and stress management and intervention-related outcomes such as practicality and cost.

**Results:**

14 studies reporting results from randomised-controlled trials were included in this systematic review. Overall, studies demonstrated mixed results in technical and non-technical skill development across a variety of procedures. However, cognitive simulation offers a practical and cost-effective learning tool that is adaptable to a variety of procedural skills programs in contemporary medical curriculum.

**Conclusion:**

This systematic review highlights the emerging potential for cognitive simulation to be adapted and integrated into standard procedural skills learning programs for medical students. However, the current evidence lacks robust insights into the efficacy of these programs due to heterogeneity of study design, specifics of the cognitive simulation program and approaches to assessment. Further research is necessary to explore the efficacy of standardised cognitive simulation programs to validate these findings.

## Introduction

Medical educators face the complex task of providing learning opportunities for the development of medical students across multiple competencies. These include technical skills such as medical knowledge, clinical interviewing and examination, diagnostic reasoning, procedural skills as well as non-technical skills such as communication, cultural competency and respect ^1^. Traditional approaches to medical education prioritise the clinical placement to develop these skills ^2, 3^. Challenges continue to remain within this paradigm of learning, particularly for procedural skills learning which is limited by issues with distractions or competing demands in the clinical environment, varying teaching expertise of supervising clinicians and inconsistent opportunities for exposure to specific procedural skills ^4– 6^.

Contemporary medical programs have shifted towards adopting adjunct experiential learning programs to provide safe and supportive procedural skills learning ^7, 8^. The purpose of these programs has been to support skills development through simulation mirroring real-life practice, therefore providing standardised learning opportunities to meet specific learning objectives of medical programs that would otherwise be difficult to achieve due to the heterogeneity of clinical placements ^8^. Despite this, medical students continue to highlight challenges with procedural skills development, including issues with negative emotions such as anxiety ^7– 9^.

In addition to physical simulation-based approaches, cognitive simulation (also known as mental imagery, mental visualisation, mental rehearsal, mental skills training, cognitive training) is becoming increasingly appreciated as a method to support procedural skills learning in both simulated and real-life settings ^10^. Central to the practice is the psychological rehearsal of tasks without physical performance ^11^. Studies have supported cognitive simulation in skills development, with durable results demonstrated across multiple disciplines including surgery, aviation, elite-level sport and music ^10, 12, 13^. Cognitive simulation has also been demonstrated to provide the co-benefits of reducing stress which proves useful to addressing the challenges experienced by trainee medical practitioners ^10, 14^. Despite this, a recent systematic review demonstrated that cognitive simulation approaches are highly heterogeneous, with no best-practice method as to their design or integration within the modern curriculum of medical schools ^15^. However, the evidence does suggest that formal cognitive simulation programs are associated with improved procedural skills for medical students ^15, 16^. This systematic review therefore aims to evaluate the efficacy and designs of current cognitive simulation training programs utilised in medical student education to identify approaches for their implementation in medical school programs.

## Methods

### Search protocol and registration

This systematic review was performed in adherence to the Preferred Reporting Items for Systematic Reviews and Meta-Analyses (PRISMA) guidelines (see supplementary material for PRISMA checklist) ^17^. The review was prospectively registered in the PROSPERO database (ID: CRD42024608479).

### Literature search

A computer-assisted comprehensive literature search was performed on Medline, Embase, CINAHL, Emcare and the World Health Organisation (WHO) International Clinical Trials Registry Platform (ICTRP) on 1 November 2024. Additional articles were screened from the reference lists of relevant articles for further inclusion. The search strategy combined keywords and medical subject headings (MeSH) terms related to cognitive simulation and medical students. The complete search strategy is available in the appendix.

### Eligibility criteria

Full-text peer-reviewed articles which evaluated the use of cognitive simulation for the procedural skills development of medical students were considered for inclusion in this systematic review. In this case, cognitive simulation is defined as any process whereby psychological rehearsal of tasks without physical performance is undertaken with or without adjunct tasks. Other terms synonymous with cognitive simulation practice include mental imagery, mental visualisation, mental rehearsal, mental skills training and cognitive training. The inclusion criteria included original randomised-controlled trials, retrospective or prospective cohort studies, retrospective or prospective case-control studies, retrospective or prospective observational studies, mixed methods studies and abstracts or conference papers reporting on such studies. Additional inclusion criteria included articles that evaluated medical students (both undergraduate and post-graduate) and implemented an educational program or curriculum that involved cognitive simulation or synonymous approaches. Articles were excluded if they were of the following study designs; reviews, meta-analyses, non-human trials, letters, opinion articles, editorials, commentaries, case reports and case series. Additional exclusion criteria included articles that evaluated students from non-medical backgrounds, implemented other educational approaches that were not of interest, had incomplete data or did not evaluate the outcomes of interest.

### Screening of the literature

Screening by title and abstract was performed by two independent investigators (KL, SL). Articles that met inclusion criteria, or have incomplete data, progressed to full-text analysis. Full-text analysis was subsequently performed independently by the same two investigators (KL, SL). Disagreement was resolved by discussion and consensus.

### Outcomes

Outcomes of interest were related to identifying the efficacy and design of cognitive simulation programs to identify approaches for medical students. These outcomes included procedural skills development of medical students following exposure to cognitive simulation educational interventions. These outcomes included proficiency (skill or expertise in a specific area), confidence, scores based on performance of procedures, technical ability (broad range of skills to perform a task), time of procedures, accuracy, precision, global rating/ overall performance, visuospatial skills, teamwork, communication / stress / anxiety. Additional outcomes of interest were related to implementation of these cognitive simulation interventions including practicality and cost.

### Data extraction

Included articles were extracted for relevant identifiers including author, year of publication, country of publication and study design. Additional parameters relevant to cognitive simulation and procedural skills were also extracted including type of cognitive simulation activity, learning activities, learning or pedagogical theory applied, duration of intervention and outcomes of interest were extracted.

### Data synthesis and statistical analysis

Statistical analysis was performed utilising Review Manager 5.4 (RevMan 5.4) software (Cochrane, London, United Kingdom). Meta-analysis was performed where homogeneous quantitative data was available for all outcomes. A random-effects model was employed if heterogeneity was deemed present, otherwise a fixed-effects model was employed. Odds ratios (OR), 95% confidence intervals (CI) and p-values were extracted or calculated from included studies. P-values were considered significant if they were less than 0.05. In the case of heterogeneous continuous data, conversion to single measures of effect was performed using the Wan method ^18^. Heterogeneity was determined using the Higgins I ^2^ test. Low heterogeneity was considered if I ^2^ was less than 25%, moderate heterogeneity if I ^2^ was between 25–50% and high heterogeneity if I ^2^ was over 50%. Meta-analyses were reported with Forest plots where possible. If homogeneous data was not available, relevant outcomes were reported descriptively.

Where qualitative or mixed methods studies were involved, data was systematically extracted based on outcomes of interest and thematically analysed using meta-synthesis. Data analysis in this way was performed independently by two investigators (KL, SL), including iterative comparison of the studies to identify and cluster recurrent themes. Furthermore, results were organised into categories and compared across the studies to identify common themes and relationships within the data.

### Risk of bias assessment

For clinical trials and observational studies, methodological rigor and quality was assessed using the Risk of Bias in Non-randomised Studies of Interventions (ROBINS-I) tool ^19^. The decision for this is that it is expected the literature examining cognitive simulation approaches would exhibit high methodological hetereogeneity and may have methodological limitations more characteristic of quasi-experimental design including unclear randomisation, lack of allocation concealment and lack of blinding. For mixed methods studies, the Mixed Methods Appraisal Tool (MMAT) was utilised ^20, 21^. In both cases, risk of bias assessment was performed by two independent investigators (KL, SL) and disagreement during this process was resolved by discussion and consensus.

### Subgroup and sensitivity analysis

Subgroup and sensitivity analysis or meta-regression was performed where possible to investigate sources of heterogeneity. Planned subgroup analyses included analysis based on whether students were in pre-clinical or clinical stages of medical school and after removal of articles at high risk of bias.

### Analysis of certainty of evidence

Certainty of evidence was assessed using the Grading of Recommendations Assessment, Development and Evaluation (GRADE) framework where possible ^22^.

## Results

### Literature search results

**Figure 1.**
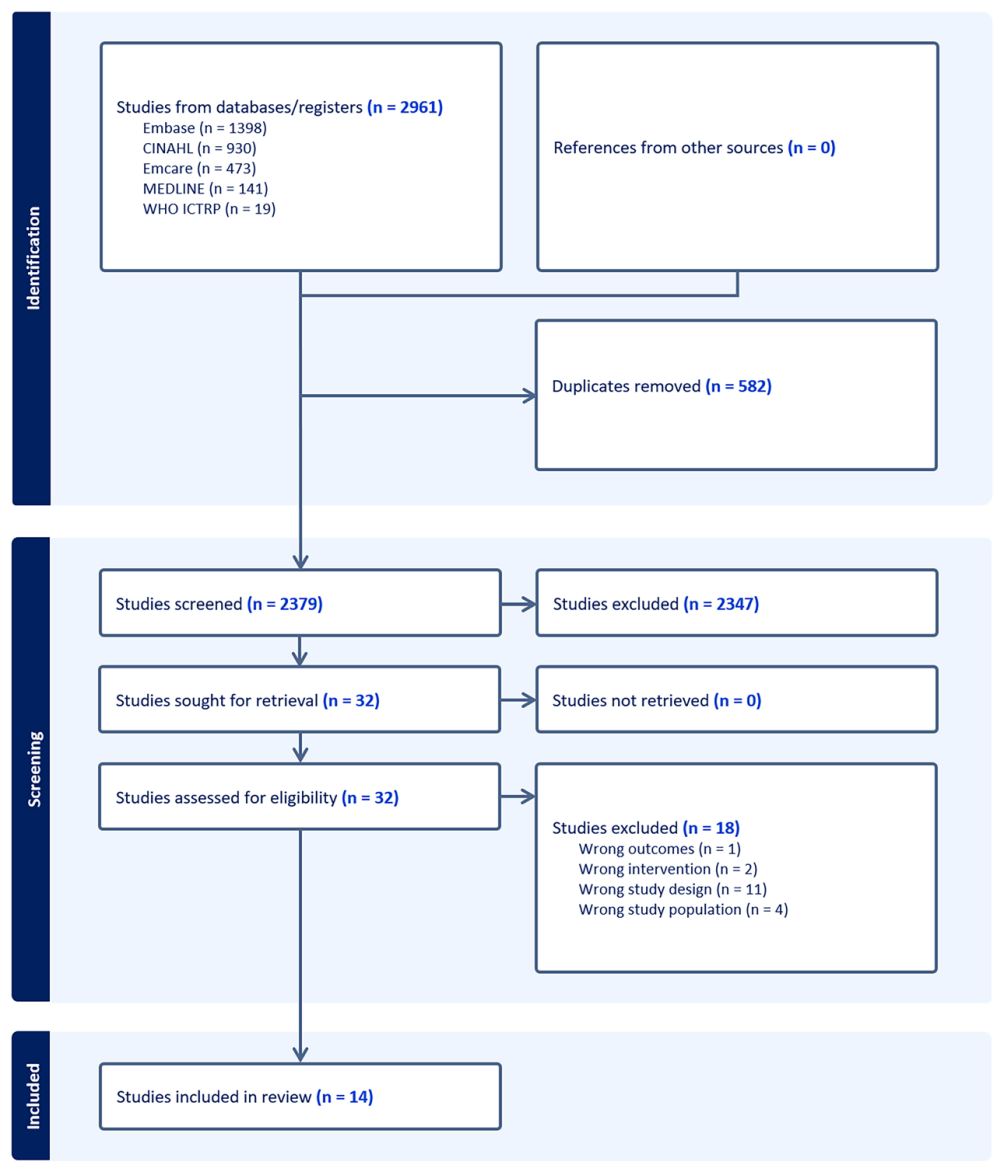
Overview of search.

A total 2961 articles were identified from the literature search. Following removal of duplicates, 2379 unique articles progressed to screening as per eligibility criteria. 32 articles progressed to full-text analysis of which 18 were excluded. The reasons for exclusion included non-concordant study design (n=11), non-concordant study population (n=4), non-concordant intervention (n=2) and non-concordant outcomes assessed (n=1). The complete search overview is presented in Figure 1.

### Overview of studies

14 unique and original studies were included in this study ^23– 36^. An overview of these included studies is presented in Table 1. Studies were published between 1983 and 2022. The most common study type was randomised-controlled trials (n=13) with the remaining study being an abstract reporting the results of a randomised-controlled trial (n=1). Studies were from diverse jurisdictions with the most common country of publication being the United States of America (USA) (n=6) and the United Kingdom (UK) (n=3). The remainder of studies were from institutions in Canada, Switzerland, Ireland, Germany and Japan (all n=1). 12 studies evaluated medical students alone, whilst one study evaluated pre-medical students and one study evaluated novice proceduralists including medical students (Stefanidis 2017) and first year junior doctors (Raison 2018) ^30, 36^. There was incomplete reporting of age, undergraduate or postgraduate status and sex of participant.

**Table 1.** Overview of included studies.

Study	Study design	Country of publication	Period of study	Sample size	Age	Study population	Undergraduate or post- graduate	Sex
Bathalon 2005	RCT	Canada	NR	44	NR	First year medical students	UG	NR
Berger-Estilita 2022	RCT	Switzerland	October 2022 - December 2022	309	21.3 +/- 1.9	First year medical students	UG	M: 120, F: 189
Cryder 2023	RCT	USA	May 2020 - October 2021	60	NR	Fourth year medical students	PG	NR
Dimitriou 2009	Abstract of RCT	Ireland	NR	57	NR	Medical students - stage NR	NR	NR
Eldred-Evans 2013	RCT	UK	NR	64	Group 1: 21.5 (20-38) Group 2: 20 (19-25) Group 3: 21 (19--34) Group 4: 21 (18-26)	Medical students - stage NR	NR	NR
Jungmann 2011	RCT	Germany	NR	40	NR	Medical students - stage NR	NR	NR
Kuriyama 2023	RCT	Japan	December 2020 - November 2021	65	Intervention: 23 (21-25) Control: 22 (21-23)	Medical students - stage NR	UG	M: 51, F: 14
Raison 2018	RCT	UK	May 2015 - August 2015 (recruitment)	64 (62 available for analysis)	Intervention: 22.3 +/- 2.5 Control: 23.8+/-3.9	Medical students with >1 year of clinical training or junior doctors	NR	M: 37, F: 27
Rakestraw 1983	RCT	USA	NR	160	NR	Second year medical students	PG	M: 108, F: 52
Sanders 2004	RCT	USA	NR	67 (65 available for analysis)	NR	Second year medical students	PG	NR
Sanders 2007	RCT	USA	March 2006 - May 2006	66	NR	Second year medical students	PG	NR
Sanders 2008	RCT	USA	NR	64	NR	Second year medical students	PG	NR
Shah 2018	RCT	UK	NR	59 (52 available for analysis)	Mental imagery: 21.6 (mean) Flashcard: 21.4 (mean) Control: 21.1 (mean)	Medical students - stage NR	NR	NR
Stefanidis 2017	RCT	USA	NR	60	Intervention: 20.5 +/-3.4 Control: 20.6+/-2.3	Pre-medical students	NA	NR

Abbreviations: RCT; randomised-controlled trial, NR; not reported, UG; undergraduate, PG; post-graduate, M; male, F; female, USA; United States of America, UK; United Kingdom

### Cognitive simulation interventions of included studies

An overview of cognitive simulation interventions of all included studies is shown in Table 2. All studies evaluated methods of cognitive simulation. These were heterogeneously defined with the most common type listed as “mental imagery” (n=8) followed by “mental practice” (n=1), “mental visualisation” (n=1), “motor imagery” (n=1), “mental practice” (n=1) “cognitive training” (n=1) and “mental skills training” (n=1). There were a wide variety of focus procedural activities with the most common being laparoscopic skills (n=3). There was significant heterogeneity in learning activity across all studies, with varying durations and methods of cognitive simulation intervention. Learning theories that were utilised to guide development of cognitive simulation programs were poorly reported. Despite this, the most common learning theories employed in the cognitive simulation interventions was dual-coding theory (n=8) ^37^. Other theories referenced in cognitive simulations include Kopta’s three stages in motor skills learning, cognitive load theory, Kolb’s experiential learning theory, theories related to neurophysiological changes and neuromodulation/neuroplasticity associated with motor imagery and Gentile’s skills acquisition mode ^38– 43^.

**Table 2.** Overview of cognitive simulation programs.

Study	Cognitive simulation terminology	Focus of activity	Cognitive simulation program activities	Underlying theory of learning	Duration of intervention	Method of assessment	Sample size of interventions
Bathalon 2005	Mental imagery	Emergency airway (cricothyrotomy; CT)	Group 1 - Kinesiology group: The 8 steps of performing a CT were shown and discussed individually including with demonstration on a mannequin. Students then perform the with supervision and immediate feedback procedure under supervision with feedback provided immediately. Group 2 - Kinesiology and mental imagery group: As per group 1 with additional 5 minutes of mental imagery training prior. In this training. Students were asked to psychologically visualise the steps before practicing the steps with paper, pen (as a knife) and fingers. Group 3 – ATLS technique (control): Performed as per guidelines.	Kopta three stages of learning motor skills Dual-coding theory	5-minute session of mental imaging skills	OSCE	Group 1: 13 Group 2: 15 Group 3: 16
Berger-Estilita 2022	Mental imagery	Intravenous cannulation	Group A: 6-minute mental imagery tutorial provided in a low stimulus environment without cannulation material provided. Group B: 6-minute simulation tutorial with arm trainer. All materials provided. Group C: 6-minute tutorial with written instructions and laminated visual aids detailing approach to intravenous cannulation.	Dual-coding theory	6-minute audio-guided mental imagery tutorial	OSCE	Group A: 105 Group B: 105 Group C: 106
Cryder 2023	Mental imagery	Central venous access	Control: Students watched a video of right internal jugular central venous cannulation with stepwise explanations. Experimental: As per control group with the addition of 6 minutes and 13 seconds of guided imagery focusing on motor, mental and psychological aspects of the procedure.	Dual-coding theory Cognitive load theory	6 minutes and 13 seconds of video-guided mental imagery	Pre and post-performance survey	Control: 33 Experimental: 25
Dimitriou 2009	Mental imagery	Laparoscopy skills (basic)	Group A: video tutorial encompassing laparoscopic skills Group B: As per group A but with the addition of a session on mental imagery. Group C: Textbook teaching with tutor supervision only.	NR	NR	Performance on haptic simulators	NR
Eldred-Evans 2013	Mental training	Laparoscopy skills (basic)	Group 1 (Control): Box-trainer practice session followed by self-practice on the same trainer. Group 2 (BT + VRS): As per Group 1 but with additional virtual reality training using VR laparoscopy simulator. Group 3 (BT+ MT): As per group 1 with the addition of a mental training program lasting 30 minutes. Group 4 (Box-free group): Practice without a box trainer. The group received a session with VR in addition to self-practice on the same VR simulator with a mental training session lasting 30 minutes.	Dual-coding theory	30-minute mental training session	Performance on box-trainer	Group 1-4: 16 each
Jungmann 2011	Mental imagery	Laparoscopic knot tying	Control: Students watched a video demonstrating laparoscopic knot tying prior to deliberate practice (knot tying 5 times at two different training sessions). Experimental: As per control group with the addition of a mental imagery training session involving a handout and demonstration video with advice on how to perform mental imagery.	Dual-coding theory	At least 3 minutes a day for at least 4 days	Performance with simulated laparoscopic knot tying	Control: 20 Experimental: 20
Kuriyama 2023	Mental visualisation	Lung auscultation	Control: Students attend a lecture on lung auscultation. Experimentation: students are taught to visualise lung sounds through 30-minute training with provision of resources such as lung sound diagrams in addition the lecture as per the control group.	Experiential learning theory	30-minute mental visualisation training	Performance with lung auscultation	Control: 30 Experimental: 35
Raison 2018	Motor imagery	Robotic urethrovesical anastomosis	Control: Students undertake basic robotic skills training on VR simulator. Students are then provided didactic instruction on urethrovesical anastomosis with a video demonstration provided prior to performing the skill. Experimentation: Same as per control group with addition of motor imagery training. Training encompasses theory of motor imagery followed by practice using a training script, then supervised practice and finally self-directed practice. Students then perform the skill.	Neurophysiological changes and neuromodulation associated with motor imagery	NR	Global Evaluative Assessment of Robotic Skills (GEARS) for technical skills. Non-Technical Skills for Surgeons (NOTSS) for non-technical skills.	Control: 29 Experimental: 33
Rakestraw 1983	Mental practice	Pelvic examination	Control: Students watch a 2-hour lecture on pelvic examination in addition to textbook readings, assignments and audiovisual resources. Experimentation (3 groups - premotor, postmotor, both): As per control however with additional two audiotapes (5 min duration each). The first audiotape assist students with forming mental images of procedural steps (premotor). The second audiotape focuses on post-practice comparison of what was done and preconceived concept of correct skill performance (postmotor).	Dual-coding theory	NR	Performance of pelvic examination	Control: 51 Premotor: 36 Postmotor: 36 Pre and postmotor: 37
Sanders 2004	Mental imagery rehearsal	Basic surgical skills	Group 1 (Control): Students undertake three suturing practice using a pig's trotter. Group 2: Students undertake two sessions of physical practice and one session on mental imagery rehearsal. Group 3: Students undertake one session of physical practice and two sessions on mental imagery rehearsal.	Dual-coding theory	30 min each, each session carried out at 1-week intervals	Live rabbit assessment and self-assessment questionnaire	NR
Sanders 2007	Mental imagery rehearsal	Venepuncture	Group 1: Students undertake 30 minutes of deliberate practice on an arm model and an additional 30 minutes of practice. Group 2: Students undertake 30 minutes of deliberate practice on an arm model arm and an additional 30-minute guided imagery and relaxation session. Group 3 (Control): Students only complete 20 minutes of deliberate practice on model arm.	NR	30 minutes each	10-item performance scale, 5-item background education scale, 6-item attitude scale and Trait Anxiety scale.	Group 1,2,3: 22 each
Sanders 2008	Mental imagery rehearsal	Basic surgical skills	Group 1 (Textbook study): Students watch a lecture on incision and suturing of pig's trotter followed by textbook study for 30 minutes. Students then undertake summative assessment of performance followed by an additional hour of supervised practice and a second summative performance. 14 days after, students undertake a second session of textbook learning prior to assessment. Group 2: Students undertake the same lecture as per Group 1 however now followed by a 30-minute guided imagery and relaxation session. Students then undertake summative assessment, supervised practice and second summative performance opportunities as per Group 1. 14 days after, students undertake a second session of guided imagery and relaxation prior to assessment.	NR	30 minutes each	Live rabbit assessment using 15-item surgical checklist, 6-rating surgical behaviour rating tool, 6-item attitude scale, 10-item prior experience questionnaire, State-Trait Inventory for adults and the Revised Minnesota Paper Form Board Test.	Group 1: 32 Group 2: 32
Shah 2018	Cognitive training or mental imagery cognitive training	Ureteroscopy	Group 1 (Control): Students undertake 20 minutes didactic ureteroscopy teaching followed by physical practice. Group 2 (flashcard): Students undertake 20 minutes of the same didactic teaching as per the control group. This is then followed by study from a set flashcards covering key points of ureteroscopy for a distal ureteric stone case. Group 3 (mental imagery): Students undertake the same didactic teaching as per the two groups above. This is followed by a mental imagery script to familiarise students with the procedure.	NR	20 minutes with additional 10 minutes to participants to familiarise themselves with the script	URO mentor simulator assessment	Group 1: 18 Group 2: 17 Group 3: 17
Stefanidis 2017	Mental skills training	Basic laparoscopic skills	Control: Students undertake nine bi-weekly small group training sessions over 5 months (45-minute FLS proficiency-based simulation in the first 3 sessions and training in the remaining 6 sessions). Mental skills training: Students undertake eight bi-weekly 45-minute mental skills education sessions (video modules and exercises) in addition to small group training as per the control group.	NR	5 minutes, bi-weekly sessions. Total of 8 sessions as part of comprehensive mental skills curriculum.	Fundamentals of Laparoscopic Suturing (FLS) for technical skills. Test Of Performance Strategies Version 2 (TOPS-2) tool for mental skills.	Control: 28 Mental skills training: 27

Abbreviations: Objective structured clinical examination; OSCE, Advanced trauma life support; ATLS, NR; not reported, BT; Box trainer, VRS; Virtual reality simulation, MT; Mental training, VR; virtual reality

### Cognitive simulation and technical procedural skills

***Standard training plus mental imagery compared to standard training alone***

Six studies compared standard training with additional MI training to standard training alone (control). Of these, three (50%) demonstrated evidence to suggest improvement in skill level with adjunct MI training compared to control. Cryder *et al.* evaluated 60 4th year medical students who were taught Central Venous Catheter (CVC) insertion and exposed to traditional teaching (educational video) with or without MI training. There was a reduction in errors and need for intervention in the MI group compared with control (Average number of errors/episodes of needing intervention were 1.29 in the experimental group compared to 2.21 in the control group, p = 0.0455) ^25^. Stefanidis *et al.* demonstrated skill improvement was greater (during the interval between initial testing and retention testing 2 months later) in the MI group compared to control when 60 medical students performed basic laparoscopic skills (average improvement of 17.8% in the mental skills group compared to 10.1% in the control group, p = 0.04) ^36^. Similarly, Bathalon *et al.* demonstrated kinesiology combined with mental imagery training (together) led to improved technical skills in cricothyrotomy compared to standard training alone (average OSCE total score (out of 25) of 20.3 in the kinesiology and mental imagery group compared to 18.2 in the control group, p<0.05) ^23^.

Conversely, the remaining three studies demonstrated lack of evidence to support technical skills development with MI. Jungmann *et al.* assessed simulated laparoscopic knot-tying in 40 medical students who were randomly assigned to either control (2 training sessions) or experimental (same 2 training sessions but with additional mental practice) groups. Skill was assessed (as "tip trajectory") in the two training sessions. Participants also completed a cube subtest of a standardised intelligence test to evaluate visuo-spatial ability. Comparison between the two groups did not demonstrate difference in skill ^28^. Kuriyama *et al.* evaluated lung auscultation skills in 65 medical students both before and after three weeks of clinical clerkship. All students attended a lecture featuring a simulator, then 35 students also received additional MI training. Auscultation skill was assessed on a simulator both at the beginning and end of the three-week period. Test scores improved in both groups, with no difference in magnitude of improvement between the two groups (p = 0.29) ^29^. Finally, Rakestraw *et al.* evaluated skills in pelvic examination of 160 medical students, who were randomised to standard training with or without additional MI training at either pre-motor stage, post-motor stage or pre and post-motor stages of skill acquisition (four groups total) which revealed no difference between the group scores ^31^.

***Mental imagery compared to alternative training strategies***

Four studies compared additional mental imagery training to other forms of additional training such as physical practice, reading written instructions, textbook study and instructional video. Two studies (50%) found MI training to be superior to instructional video and textbook study respectively. Raison *et al.* evaluated urethrovesical anastamosis in 62 novice surgeons randomised to either receive standard teaching (basic skills course plus instructional video) with motor imagery training, or standard teaching with instructional video reinforcement. They demonstrated higher mean performance scores in the motor imaging group (13.1 vs 11.4, p = 0.03) ^30^. Sanders *et al.* compared basic surgical skills of 66 medical students following standard training with MI or additional textbook study. While there was no significant difference between the two groups on the first two assessments (performed immediately following standard training and the first session of the allocated intervention), the mental imagery group performed significantly better in the final assessment than the textbook study group ^32^.

The remaining two studies (50%) found MI training to be equivalent to the evaluated alternative training techniques (physical practice and reading written instructions). Berger-Estilita *et al.* assessed intravenous cannulation (IVC) skills in 309 1st year medical students, immediately after a 6 min randomised self-learning refresher (6 months after their initial 4-hour tutorial) of either MI audioguide, physical practice or reading written instructions. Students were evaluated using a 15-item standardised checklist, and scores were compared between groups, with no difference in performance between these groups ^24^. Sanders *et al.* compared MI to additional physical practice in the suturing skills development of 65 medical students. Students were randomised to receive either three sessions of physical practice, two sessions of physical practice and one session of MI rehearsal, or one session of physical practice and two sessions of MI rehearsal. When skills were evaluated in live rabbit surgery after the completion of all training sessions, there was no difference in skill level between the three groups ^32^.

***Mental imagery compared to alternative training strategies and to control***

Four studies were designed to compare MI training to control and to alternative training methods. Two of these studies (50%) found no difference between all groups (control, MI and alternative training). Dimitriou et al evaluated laparoscopic skills (via simulator) in 57 medical students randomised to one of 3 groups - video tutorial, video tutorial plus relaxation/MI rehearsal, and textbook teaching. Laparoscopic skills were assessed using a haptic simulator both one day before and one day after the allocated intervention, with all three groups demonstrating improvement between the two assessments. However, there was no difference in performance between the three groups ^26^. The only significant finding by the authors was that the MI rehearsal group showed greater improvement in ambidexterity ^26^. Similarly, Shah *et al.* compared simulation alone (control), simulation with additional flashcards and simulation with MI training on ureteroscopic skills of 59 medical students. Following initial didactic teaching and simulator training (all groups) participants practiced on a URO mentor simulator, before undergoing their allocated intervention. After an average of ten days (5–21 days), participants returned to complete another assessed task on the simulator. Performance reports generated by the simulator were extracted and compared between groups. These comprised data pertaining to a variety of parameters including time taken, trauma, catheterisation attempts, fragmentation, total laser energy, laser misfires, maximal stone extracted/residual and x-ray exposure time. When the groups were compared, the only parameter which reached statistical significance was laser misfires, with a significant reduction in misfires (as a percentage of total laser fires) seen in the MI group (0.83% compared to 8.67% in the control group and 6.85% in the flashcard group) ^35^.

The remaining two studies (50%) found the MI group to be superior to control. Sanders *et al.* assessed venepuncture skill of 66 medical students randomised into one of three groups: control, physical practice and MI. All participants received an initial lecture-demonstration and 30 minutes of guided venepuncture practice on artificial arms. One group received an additional 30 minutes of such practice, the next received a 30-minute session of guided imagery, and a control group received no additional training. Venepuncture skills (performed on human arms) were then assessed by trained physician educators (score out of 60). The authors found no difference between the guided imagery group (mean 44.15, range 31–53) and additional physical practice group (mean 44.89, range 27–55), however both groups performed significantly better than control (mean 39.57, range 29–55) ^33^. Eldred-Evans *et al.* evaluated four different training regimes for teaching laparoscopic skills (cutting a circle) to medical students. 64 participants were randomised to either box-trainer alone (BT), box-trainer plus virtual reality simulator (VRS enhanced), box-trainer plus mental training (MT enhanced) or virtual reality simulator plus mental training (box free). The authors demonstrated that the VRS-enhanced group scored best in all domains (precision, accuracy and overall performance) when compared to control, with the MT-enhanced group demonstrating the next highest scores in precision, accuracy and overall performance ^27^.

***Mental imagery and procedural time***

Five studies assessed the impact of mental imagery training on time taken to perform the relevant skill. Four of these found no significant difference between experimental and control groups. Bathalon *et al.* found no difference in time taken to perform cricothyrotomy in control (taught using advanced trauma life support technique), kinesiology or kinesiology and MI groups ^23^. Cryder *et al.* found no difference in time taken to insert a CVC after traditional teaching (educational video) with or without MI ^25^. Jungmann *et al.* found no difference in laparoscopic knot tying time between 40 medical students exposed to standard training (two training sessions, control group) with or without additional mental practice ^28^. Similarly, Shah *et al.* demonstrated no difference in timing of ureteroscopy in groups exposed to simulation training only, simulation with the addition of flashcard training or simulation with mental imagery training ^35^. Despite this, Eldred-Evans *et al.* revealed laparoscopic circle-cutting skills were slowest in the MT-enhance group (06:44), followed by the box-free group (06:02), with both again significantly slower than control (05:39) and VRS-enhanced (04:55) groups ^27^.

### Cognitive simulation and non-technical skills

***Mental imagery and procedural confidence***

Two studies explored confidence levels with MI compared to control interventions. Cryder *et al.* explored self-rated confidence in CVC placement on a scale of 1–10 at three points during the study: firstly prior to watching the instructional video, next after watching the video (and participation in guided MI for the intervention group), and finally after placing the CVC. Confidence improved across the course of the study for both groups. Despite this, the authors reveal lack of evidence to suggest improved confidence between MI compared to control groups ^25^. On the other hand, Kuriyama *et al.* assessed student self-efficacy in lung auscultation (including perceived ability and confidence) prior to and at the completion of four weeks of clinical clerkship. The authors revealed improved subjective confidence ratings overall in both groups, with greater improvement seen in the MI group compared to control (adjusted mean difference between pre-and post-questionnaire, 1.7 vs. 1.3, p = 0.020) ^29^.

***Mental imagery and capacity to deal with stress***

Two studies explored the impact of MI interventions on stress outcomes. Stefanidis *et al.* explored the impact of a mental skills curriculum when teaching laparoscopic skills on subjective (6-item State-Trait Anxiety Inventory; STAI-6) and objective (heart rate measurements) stress. All participants first completed a laparoscopic skills curriculum spanning approximately 5 months, during which time the intervention group were also participating in a mental skills curriculum, which provided coaching in mental imagery among other mental skills. Participants subsequently had their stress levels assessed in a simulation three weeks after their training was completed (transfer test) and again two months later (retention test). The mental skills group reported lower levels of subjective stress (based on STAI-6 responses) both during the transfer test (11.6 vs. 13.7, p = 0.05) and the retention test (11 vs. 12.7, p = 0.12) although the latter was not statistically significant ^36^. Interestingly, during the retention test the control group had significantly lower baseline-adjusted heart rate than the mental skills group (109.2 vs. 120.9, p = 0.04), suggesting higher levels of physiological stress in the latter ^36^. Raison *et al.* assessed urethrovesical anastamosis ability while responding to increasingly stressful distractor events ranging from engaging in simple conversation with team members to the simulated patient becoming haemodynamically unstable. Performance was graded by a non-technical skills expert using the Non-Technical Skills for Surgeons (NOTSS) behavioural rating system. When mean NOTSS scores were compared, there was no significant difference between the control group (26.4) and motor imagery group (25.8, p =0.77) ^30^.

### Mental imagery skills acquisition

Three studies explored MI skills development. Raison *et al.* used a revised movement imagery questionnaire (MIQ) to judge the quality of motor imagery skill. When MIQ scores were compared between groups, the motor imagery group was found to have a significantly higher overall mean score than control (5.11 vs. 4.46, p = 0.03) ^30^. Shah *et al.* assessed the effectiveness of MI training using a questionnaire provided only to members of the intervention group at the completion of the study. The questionnaire comprised of four questions exploring how easily participants could "see" the procedure, how vivid their mental images were, how easily they could "feel" the procedure, and their ability to explain the steps required to perform the procedure. Mean response scores (out of 10) for each question were 7.71, 7.9, 6.7 and 7.7 respectively, demonstrating that participants felt reasonably confident in their mental imagery skills ^35^. Stefanidis *et al.* included evaluations of mental skills ability using the Test of Performance Strategies Version 2 (TOPS-2) self-report instrument which assessed a range of psychological skills, including MI. The post-intervention TOPS-2 imagery-performance (subsection) scores were significantly higher in the mental skills curriculum (MSC) group (4.0) compared to control (3.6, p = 0.03) ^36^. Furthermore, the overall TOPS-2 scores in the MSC group also improved significantly compared to control across the course of the study (up 2.2% vs. down 4.6%, p = 0.008) demonstrating that the mental skills training provided to the intervention group was effective ^36^.

### Perceived utility of mental imagery

Two studies evaluated the perceived utility of MI interventions. Stefanidis *et al.* demonstrated that of the students in the MSC group who completed the post-intervention survey (54%), at least 83% of respondents felt their ability was enhanced with the MSC and 85% expected to use these improved skills to better manage future stressful scenarios ^36^. Additionally, Rakestraw *et al.* in their evaluation of pelvic examination, asked participants to rate all the learning modalities provided (scale of 1 (no help) to 7 (very helpful)) and rank them in order of utility. The students assigned to one of the three intervention groups overall rated the mental imagery audiotapes as more useful than the introductory lecture and text reading, but less useful than observation of peer practice, demonstration and practice on a model and practice on a patient. Average ratings of MI utility (out of 7) were 5.11 for the pre-motor tape group, 5.11 for the post-motor tape group and 5.35 for the pre and post-motor group ^31^.

### Cost-effectiveness of mental imagery interventions

Three studies discussed the expected cost-effectiveness of MI intervention, although no study performed a formal cost-effectiveness analysis. All three agreed that MI training generated little cost, including minimal ongoing costs once supportive materials have been developed ^24, 27, 35^.

### Risk of bias

**Figure 2.**
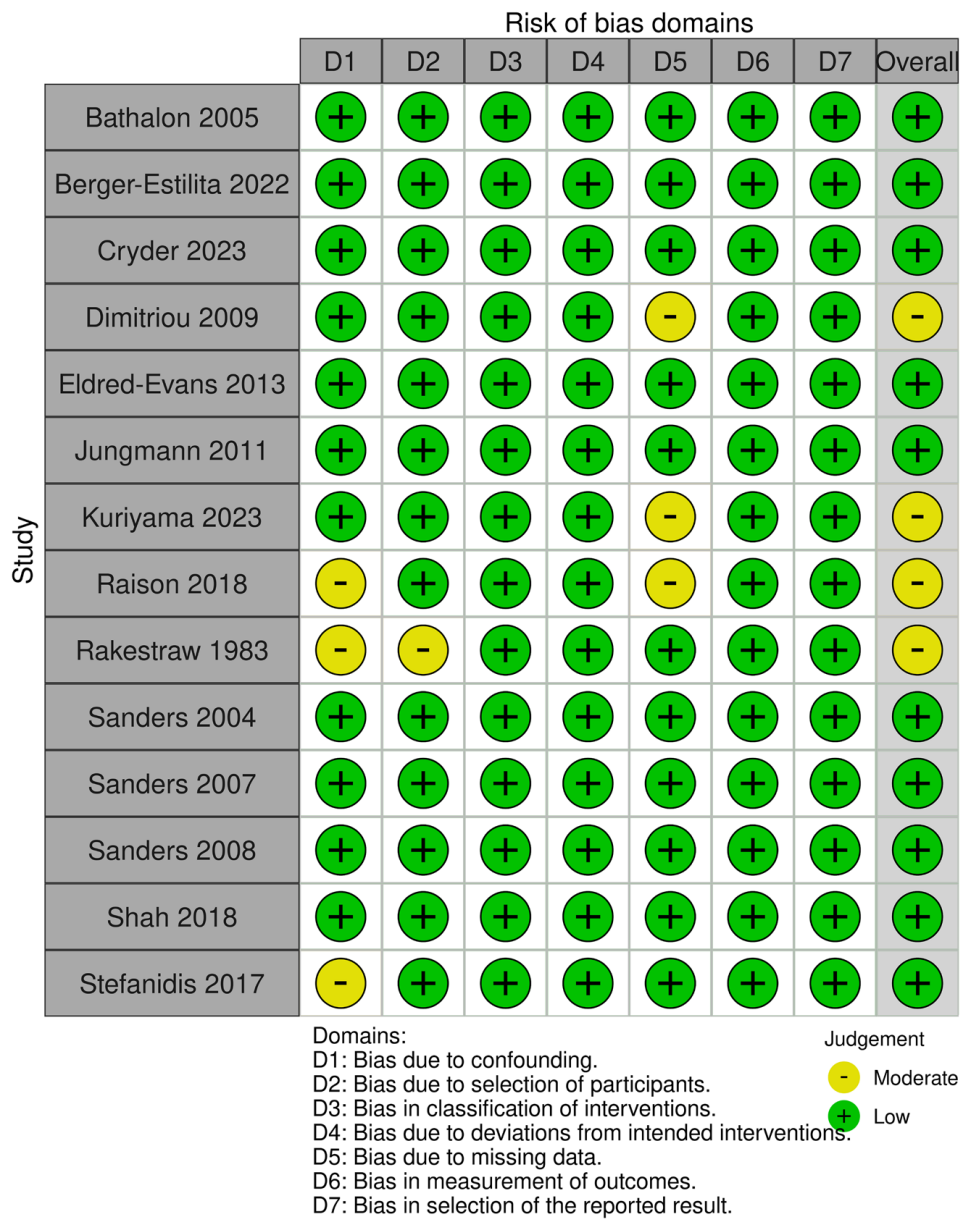
Risk of bias assessment utilising the ROBINS-I tool.

Overall, articles were generally of low (n=10) to moderate (n=4) risk of bias ( Figure 2). Key areas that limited methodological rigour included biases related to confounding due to varying expertise of the underlying cohort, incomplete reporting of participant selection and missing data due to excluded potential participants. No risk of bias assessment was performed using the MMAT as there were no mixed methods studies.

## Discussion

This systematic review highlights variable outcomes of cognitive simulation, when implemented as an adjunct to traditional approaches of teaching, in improving the technical and non-technical procedural skills of medical students. While several studies highlight improved technical skills across a variety of surgical and non-surgical procedure as well as non-technical attributes in confidence and stress management, overall, there were mixed results due to the limitations of the underlying evidence. In particular, the heterogeneity of study design, cognitive simulation program and methods of procedural skills assessment limited the ability to draw robust conclusions about the efficacy of cognitive simulation methods for procedural skills development. Furthermore, statistical methods to draw more robust conclusions such as meta-analysis, as well as our planned subgroup and sensitivity analyses were not possible given the variability in outcome reporting. Despite these limitations, the included studies highlight key advantages of cognitive simulation approaches such as cost efficiency, accessibility, versatility and adaptability of this learning approach.

Cognitive simulation approaches have shown benefits in improving skills in various disciplines, such as elite sport, aviation and music ^12, 13, 44^. The practice incorporates various learning theories. Central are dual-coding theory, where learning is suggested to occur via a combination of verbal code (language) and non-verbal code (mental imagery), as well as cognitive load theory, which suggests that learners who are able to imagine a concept (or procedure) perform complex tasks with greater proficiency due to consolidation of information ^45, 46^. This approach therefore lends itself well to tasks that require multiple steps or are inherently complex. Surgical specialties in particular have adopted cognitive simulation as an adjunct to surgical skills development, with durable improvement in technical skills across a range of surgical specialties in addition to the co-benefits of stress reduction ^10, 47^. Furthermore, studies suggest these benefits extend outside of surgical specialties, with evidence that cognitive simulation can improve resuscitation efficacy as per the Advanced Trauma Life Support (ATLS) algorithm ^48^. Despite this, implementation of cognitive simulation practices in healthcare generally occurs with great variability and in a non-standardised manner, which makes it difficult to understand whether these practices may also be translated with good efficacy to other areas of medicine such as in medical student education. This heterogeneity also affects the ability for robust analysis of the efficacy of these programs in relation to improving the procedural skills of medical students. Our systematic review highlights various parallels to the current literature, including the highly varied cognitive simulation program designs and heterogeneity of the underlying literature. Despite this, our results suggest there may be a role for standardised cognitive simulation to support current procedural skills development programs and teaching, however further research is required with a focus on best-practice principles in the design and integration of these interventions.

On evaluation of the included studies, there are several key considerations that are gained that would significantly impact efforts of incorporating cognitive simulation into medical education. In particular, the current evidence evaluates procedural skills that arguably may be considered outside the scope of competency of medical students and junior doctors. Notably, Shah *et al.* evaluate student performance on ureteroscopy, Raison *et al.* evaluate formation of urethrovesical anastomoses and Bathalon *et al.* evaluate skills in performing a surgical cricothyrotomy ^23, 30, 35^. These study designs may mask the true effect of cognitive simulation due to inherent confounding variables based on difficulty of the evaluated task. In addition to the highly variable cognitive simulation practices implemented and the varying methods of assessment, it is difficult to robustly characterise the true efficacy of this approach for medical students. Future research should prioritise evaluating cognitive simulation in the context of tasks appropriate for the level of learner training.

Following this, there is a need for more standardised cognitive simulation program design. At present, given the infancy of this approach in the medical curriculum, it is understandable that there is a lack of a best-practice approach or standardised method via which these practices are implemented. Furthermore, there are structural and skill-based challenges towards implementation, including the need to train educators on cognitive simulation theory and practice as well as the need to develop resources to support both training of educators and students. This approach aligns with recommendations for optimal simulation-based education program design, deliberate practice and skills acquisition, facilitator training and curriculum integration ^1^. Importantly, despite educators highlighting the cost-effectiveness of cognitive simulation, it is likely these initial steps to forming the foundations of the practice will require an investment of resources and time. Given this, it is important for educators, medical schools and students to “buy-in” to these approaches. The literature suggests that this process of implementation is complex and requires important steps of faculty and student consultation, program design, piloting and integration ^15^. Furthermore, key considerations to implementing these programs include aligning learning outcomes and goals with the intended cognitive simulation strategy and ensuring these programs are designed in line with the resources that are available to the jurisdiction (consumables, staffing, expertise levels) ^15^. It is also clear from our results that cognitive simulation techniques can be learned and developed for students, and therefore faculty should consider programs that not only use these skills for procedural learning but also seek to develop these skills in their own right. For resource poor settings or low-middle income countries (LMICs) however, the cost advantage of providing cognitive simulation programs may be appealing, albeit requiring substantial initial investment (both cost and time) to design. Approaches to scale these programs in such settings may include adopting and adapting programs from similar or neighbouring jurisdictions. Furthermore, in the landscape of new and emerging technologies such as generative artificial intelligence, the use of large language models may break down the barriers to developing these programs such as by streamlining the process of program design, program implementation tips and evaluation ^49^. Despite this, faculty should be conscious of the limitations of these tools, such as hallucination and lack of validation, thereby highlighting the importance of the verification process by experiences medical educators ^49^.

This systematic review to the authors’ knowledge is the first to evaluate the effects of cognitive simulation programs on the procedural skills education of medical students. Importantly, there are limitations of this review to be considered. Firstly, two studies included pre-medical or junior doctor participants however were still analysed given the expected level of technical expertise between cohorts was similar ^30, 36^. Specifically, we understand the capabilities and skills within the medical student cohort to be highly variable. For example, a new first year medical student and a final year premedical student or a final year medical student and a new medical graduate may have a similar skillset but differ only by title. This however does represent an *a priori* exclusion. Moreover, given the heterogeneity of underlying studies and paucity in data, it was not possible to perform a meta-analysis of all our outcomes of interest, nor perform our intended subgroup and sensitivity analyses. In particular, this heterogeneity stems from the variability in cognitive simulation program design, which likely is related to the resources and skillsets available to each jurisdiction. Furthermore, another explanation for this variability is that it is also likely these programs were designed specifically for the respective jurisdictions and learning needs of students. In this way, we were also not able to pool data to analyse the overall cost-effectiveness of cognitive simulation programs for medical students. Importantly, some skills assessed by certain studies may be considered outside the scope of practice or competence of medical students (such as cricothyrotomy and ureteroscopy) and therefore may be considered confounders. Finally, the underlying studies did not evaluate long-term skills retention due to the inherent program and assessment design and therefore the effect of cognitive simulation on sustainable procedural skills development remains unknown.

## Conclusion

This systematic review identified cognitive simulation as a promising but preliminary practical, cost-effective and adaptable learning strategy that can be incorporated into medical education programs for procedural skills development. The evidence at this stage demonstrates mixed results with respect to technical skill and non-technical skills improvement, with limitations attributed to the heterogeneity of studies. There is a need for more standardised theory-informed cognitive simulation programs to be designed and research particularly looking at fidelity checks and long-term retention outcomes to validate the true effect of this learning approach on medical student procedural skills training.

## Data Availability

The data for this manuscript was derived from open-access publicly available peer reviewed academic papers which were appropriately cited. Open Science Framework (OSF). Cognitive simulation for the procedural skills learning of medical students: A systematic review. DOI 10.17605/OSF.IO/N3V95 ^50^. OSF | Cognitive simulation for the procedural skills learning of medical students: A systematic review. This project contains the following underlying data: PRISMA Checklist. (Word document file of PRISMA checklist for systematic review) PRISMA flowchart. (TIFF image file of PRISMA flowchart) Data is available under the terms of the CC0 license.
