# Supplementary material for: Cognitive simulation for the procedural skills learning of medical students: A systematic review
Source: MedEdPublish (2016). 2025 Nov 16;15:181. Originally published 2025 Oct 2. [Version 2] doi: 10.12688/mep.21205.2 (PMC12824485; doi:10.12688/mep.21205.2)
Supplement: Peer review history

# Reviewer report: Reviewer response for version 2

**DOI:** 10.21956/mep.22914.r44570

**Published:** 2026-01-21

**Authors:** Rhoda Meyer

**Affiliations:** 1 Stellenbosch University, Cape Town, Cape Town, South Africa

Thank you for the opportunity to review this article titled: Cognitive simulation for the procedural skills learning of medical students: A systematic review.

 This systematic review examines cognitive simulation interventions for developing procedural skills in medical students. The authors identified 14 studies (primarily RCTs) with mixed results regarding technical and non-technical skill improvements.

 The abstract offers a definition of the concept, provides a brief description of the methods and offers a summary of the results. The link between the gap and the need for the review, however, is not clear, for example, how does variability in practices warrant an exploration into the efficacy and value of cognitive simulation. In addition, the envisaged contribution of this review to HPE practice is not evident.

 Introduction

 The introduction provided a good background to the study, highlighting the shift toward safer learning environments. There is a strong motivation to move beyond traditional simulation to cognitive simulation. However, some arguments are not clear, for example, it is mentioned that learning during clinical placement is a paradigm – the authors should explain clearly what is meant by paradigm in this context. While one must acknowledge that learning in the clinical environment is challenging, it is important to recognise that this environment also offers authentic learning experiences that have the potential to move the student beyond developing just clinical skills, to developing competence and professional identity.

 It is mentioned that cognitive simulation is becoming increasingly appreciated as a method for procedural skills. However, it is not clear how this skill development is different to the traditional way of developing these skills. In other words, what value does it offer HPE?

 Methodology

 The authors speak about evaluating on the one hand and implementation guidance on the other. These require different methodologies, and thus the authors need to clarify whether they want to look at the effectiveness of implementation or both and refine the research question accordingly.

 In terms of the literature search, a concern is that there is no indication of whether a librarian or information specialist was consulted when creating the search string and keywords. This raises concerns about the robustness of the search. It is also not clear how the authors decided on which databases to include. A brief justification is needed.

 The authors explain the screening process in a logical manner. However, certain aspects require clarification, for example, why were articles that had incomplete data included in the full-text analysis?  Under data synthesis, it is not clear what the authors mean by ‘thematically analysed’. A justification for this choice and a description of this process are needed.

 Discussion

 The discussion does not adequately address why the results were so mixed. In addition, practice guidance for educators wanting to implement these programmes is lacking. The study highlights the heterogeneity and mixed evidence for cognitive simulation in medical education; however, the manuscript needs substantial revision to meet indexing standards for a systematic review.

Are the rationale for, and objectives of, the Systematic Review clearly stated?

Partly

Is the statistical analysis and its interpretation appropriate?

Not applicable

Are sufficient details of the methods and analysis provided to allow replication by others?

Partly

Are the conclusions drawn adequately supported by the results presented in the review?

Partly

Reviewer Expertise:

Health professions education, qualitative methodology, faculty development, clinical learning

I confirm that I have read this submission and believe that I have an appropriate level of expertise to confirm that it is of an acceptable scientific standard, however I have significant reservations, as outlined above.

# Reviewer report: Reviewer response for version 2

**DOI:** 10.21956/mep.22914.r45029

**Published:** 2026-01-21

**Authors:** Gerald M Fried, Junko Tokuno

**Affiliations:** 1 McGill University Faculty of Medicine and Health Sciences (Ringgold ID: 12367), Montreal, Québec, Canada

The manuscript titled “Cognitive simulation for the procedural skills learning of medical students: a systematic review” brings the concept of cognitive simulation to the readers’ attention. Whereas mental imagery is widely used in preparation for performance in many fields, such as arts and sports, evidence of its value in procedural training in medicine has not been thoroughly explored. The current paper provides interesting insights, but there are serious limitations.

 Fundamentally, to use mental imagery in preparation for performance, one must have a clear mental model to use. Visualization is a strategy used widely by elite performers. Since the study addresses its application for medical students, who one might assume are relatively naïve learners for many of the procedures reviewed, the incorporation of cognitive simulation should be built on some foundational education about the procedure such as through an online module, videos or lectures. This should be an important element in the discussion in this paper. It would be very helpful for the reader to understand whether medical students are just too junior to benefit from cognitive simulation, or at least what is the optimal foundational training to prepare the learner for this strategy.

1. In Introduction, authors introduced “a recent systematic review”, but this is a narrative review by the first author (ref 15). Please correct.
2. In Methods, eligibility criteria can be improved with clear descriptions about timeframe and languages included for the search. In addition, it would be important to have a detailed definition for the population included in this study is expected. Although this review seems to focus on studies for medical students, “post-graduate” students and “junior doctors” are included in Methods and Results.
3. Standardizing the terminology for cognitive simulation will improve the readability of the manuscript. Although “cognitive simulation” is used in most parts, the authors also use mental imagery extensively in the review, including as sub-headings for results. The abbreviation “MI” for mental imagery should be clearly stated I before it is used.
4. Cognitive simulation may be used by learners even if it is not explicitly taught or requested in these trials. This may be another limitation in some of the papers in the review.
5. The findings in the Results do not support the authors’ conclusions that cognitive simulation is cost-effective. Although cognitive simulation itself can be delivered with little cost, but evidence of its effectiveness in improving performance, especially in this novice learner population, is very limited.

Are the rationale for, and objectives of, the Systematic Review clearly stated?

Yes

Is the statistical analysis and its interpretation appropriate?

Partly

Are sufficient details of the methods and analysis provided to allow replication by others?

Partly

Are the conclusions drawn adequately supported by the results presented in the review?

Partly

Reviewer Expertise:

Medical education, surgery, simulation

We confirm that we have read this submission and believe that we have an appropriate level of expertise to confirm that it is of an acceptable scientific standard, however we have significant reservations, as outlined above.

## References

[1] : A Process Evaluation of a Performance Psychology Intervention for Transitioning Elite and Elite Musicians. *Frontiers in Psychology* .2020;11: 10.3389/fpsyg.2020.01090 10.3389/fpsyg.2020.01090 32670138 PMC7326055

[2] : The efficacy of psychological skills training for enhancing performance in sport: a review of reviews. *International Journal of Sport and Exercise Psychology* .2024;22(4) : 10.1080/1612197X.2023.2168725 1012-1029 10.1080/1612197X.2023.2168725

[3] : The benefits of guided imagery on athletic performance: a mixed-methods approach. *Frontiers in Psychology* .2025;16: 10.3389/fpsyg.2025.1500194 10.3389/fpsyg.2025.1500194 PMC1202189040290535

[4] : Visual Performance and Sports: A Scoping Review. *Journal of Sport and Exercise Psychology* .2024;46(4) : 10.1123/jsep.2023-0267 205-217 10.1123/jsep.2023-0267 38815973

# Reviewer report: Reviewer response for version 2

**DOI:** 10.21956/mep.22914.r44432

**Published:** 2025-12-10

**Authors:** Syeda Sadia Fatima

**Affiliations:** 1 Aga Khan University, Karachi, Karachi, Pakistan

No further comments.

Are the rationale for, and objectives of, the Systematic Review clearly stated?

Yes

Is the statistical analysis and its interpretation appropriate?

Partly

Are sufficient details of the methods and analysis provided to allow replication by others?

Partly

Are the conclusions drawn adequately supported by the results presented in the review?

Yes

Reviewer Expertise:

NA

I confirm that I have read this submission and believe that I have an appropriate level of expertise to confirm that it is of an acceptable scientific standard.

# Reviewer report: Reviewer response for version 1

**DOI:** 10.21956/mep.22712.r43840

**Published:** 2025-10-31

**Authors:** Syeda Sadia Fatima

**Affiliations:** 1 Aga Khan University, Karachi, Karachi, Pakistan

This manuscript systematically reviews cognitive simulation (CS) where fourteen studies are included. Results are mixed: several trials show improved technical outcomes or selected nontechnical outcomes (e.g., confidence, stress), while others find no difference versus control or alternative training. Several methodological and reporting issues must be addressed to ensure scientific robustness and interpretability.

**Comments **

- Methods state that RevMan was used, and meta-analyses/forest plots were planned; pooled results are not presented, and it remains unclear precisely which outcomes were too heterogeneous to combine.
- Revise Methods to specify *a priori* which primary/secondary outcomes were eligible for pooling.
- In Results, explicitly list outcomes that could not be pooled and why (construct heterogeneity, incomparable scales, insufficient data).
- Provide a clear operational definition of CS and a terminology map that links each study’s label to your definition (e.g., guided imagery script, audio-guided MI, combined MI+kinesiology).
- ROBINS-I is intended for non-randomized studies. The figure is referenced without domain-level narrative, and no inter-rater reliability is reported. Use RoB 2 for RCTs and ROBINS-I for non-randomized studies, report tool choice per study. Add a brief narrative of domain-level risks (randomization, deviations, missing data, measurement, reporting) and inter-rater agreement (e.g., Cohen’s κ or percent agreement) and how disagreements were resolved.
- State clearly whether Subgroup/sensitivity analyses were impossible; if so, provide a qualitative subgroup comment (e.g., trends by learner stage, surgical vs non-surgical tasks). If feasible, conduct sensitivity analyses excluding high-risk studies and report any changes in direction/magnitude of effects.
- Temper language to “promising but preliminary,” emphasize heterogeneity and the need for standardized, theory-informed CS protocols with fidelity checks and long-term retention outcomes.
- Also focus on the applicability and feasibility in LMIC setting 

Are the rationale for, and objectives of, the Systematic Review clearly stated?

Yes

Is the statistical analysis and its interpretation appropriate?

Partly

Are sufficient details of the methods and analysis provided to allow replication by others?

Partly

Are the conclusions drawn adequately supported by the results presented in the review?

Yes

Reviewer Expertise:

technology enhanced education, AI related education reforms.

I confirm that I have read this submission and believe that I have an appropriate level of expertise to confirm that it is of an acceptable scientific standard, however I have significant reservations, as outlined above.

# Response

**Published:** 2025-10-31

**Authors:** Khang Duy Ricky Le

**Affiliations:** School of Medicine, Deakin University Faculty of Health, Geelong, Victoria, Australia

Dear MedEdPublish editorial office,

 It is with great privilege that we submit our revised version of our manuscript titled “Cognitive simulation for the procedural skills learning of medical students: A systematic review.” for review. We have judiciously attended to the reviewer comments, which for orientation are provided verbatim below alongside our replies. This is supported by a revised copy of our manuscript with tracked changes.  

**Methods state that RevMan was used, and meta-analyses/forest plots were planned; pooled results are not presented, and it remains unclear precisely which outcomes were too heterogeneous to combine.**

 We thank the reviewer for their comment. This was made clear in our discussion that no outcomes of interest were possible in the form of statistical meta-analysis. The wording was changed to *“Moreover, given the heterogeneity of underlying studies and paucity in data, it was not possible to perform a meta-analysis of all our outcomes of interest, nor perform intended subgroup and sensitivity analyses.” *to make this clearer in the limitations area of the discussion.

**Revise Methods to specify *a priori* which primary/secondary outcomes were eligible for pooling.**

 We thank the reviewer for their comment. Classically for meta-analyses, all outcomes are eligible for analysis if there is enabling data. This has been made clearer in the data synthesis and statistical analysis section of the methods as follows *“Meta-analysis was performed where homogeneous quantitative data was available for all outcomes.”*

**In Results, explicitly list outcomes that could not be pooled and why (construct heterogeneity, incomparable scales, insufficient data).**

 We thank the reviewer for their comment. Typically these points are discussed in limitation sand so have been raised in the limitations area of the discussion as follows *“Moreover, given the heterogeneity of underlying studies and paucity in data, it was not possible to perform a meta-analysis of all our outcomes of interest, nor perform intended subgroup and sensitivity analyses.”*

**Provide a clear operational definition of CS and a terminology map that links each study’s label to your definition (e.g., guided imagery script, audio-guided MI, combined MI+kinesiology).**

 We thank the reviewer for their comment. We have amended the eligibility criteria to provide a definition of cognitive simulation as follows *“In this case, cognitive simulation is defined as any process whereby psychological rehearsal of tasks without physical performance is undertaken with or without adjunct tasks.” *This is also known as “ *mental imagery, mental visualisation, mental rehearsal, mental skills training, cognitive training* *” *which is referred to in the introduction but also added to the methods.

**ROBINS-I is intended for non-randomized studies. The figure is referenced without domain-level narrative, and no inter-rater reliability is reported. Use RoB 2 for RCTs and ROBINS-I for non-randomized studies, report tool choice per study. Add a brief narrative of domain-level risks (randomization, deviations, missing data, measurement, reporting) and inter-rater agreement (e.g., Cohen’s κ or percent agreement) and how disagreements were resolved.**

 We thank the reviewer for their comment. We chose ROBINS-I as the tool, acknowledging the intent and original purpose of the tool for multiple reasons. For example, the tool has the  capacity to systematically appraise intervention studies with heterogeneous methodological features which we expected to see as part of this systematic review. This is relevant as although most included studies were labelled as randomised controlled trials, several demonstrated methodological limitations more characteristic of quasi-experimental design including unclear randomisation, lack of allocation concealment and lack of blinding. The methods have been amended to reflect this decision making. Disagreement during the process of risk of bias assessment was resolved by consensus and this was discussed in the methods section, therefore no changes have been made related to this, particularly related to inter-rater agreement as there were no major conflicts.

**State clearly whether Subgroup/sensitivity analyses were impossible; if so, provide a qualitative subgroup comment (e.g., trends by learner stage, surgical vs non-surgical tasks). If feasible, conduct sensitivity analyses excluding high-risk studies and report any changes in direction/magnitude of effects.**

 All sensitivity and subgroup analyses as discussed in the methods section a priori were not possible. This is mentioned in the limitations section of the discussion and the planned subgroup and sensitivity analyses are defined in the methods section.

**Temper language to “promising but preliminary,” emphasize heterogeneity and the need for standardized, theory-informed CS protocols with fidelity checks and long-term retention outcomes.**

 We have amended the language as suggested in the conclusion.

**Also focus on the applicability and feasibility in LMIC setting**

 We thank the authors for the comment, although LMIC was not explicitly stated, there is a paragraph discussing implementation and challenges related to this with resource poor settings. I have changed this to include LMICs as well.
